# An Enriched European Eel Transcriptome Sheds Light upon Host-Pathogen Interactions with *Vibrio vulnificus*

**DOI:** 10.1371/journal.pone.0133328

**Published:** 2015-07-24

**Authors:** Agnès Callol, Felipe E. Reyes-López, Francisco J. Roig, Giles Goetz, Frederick W. Goetz, Carmen Amaro, Simon A. MacKenzie

**Affiliations:** 1 Departament de Microbiologia i Ecologia, Universitat de Valencia, Burjassot, Spain; 2 Institut de Biotecnologia i Biomedicina, Universitat Autònoma de Barcelona, Bellaterra, Spain; 3 Department de Biologia cel·lular, Fisiologia Animal i Immunologia, Universitat Autònoma de Barcelona, Bellaterra, Spain; 4 Northwest Fisheries Science Center, Seattle, United States of America; 5 Institute of Aquaculture, University of Stirling, Stirling, United Kingdom; University of Tuscia, ITALY

## Abstract

Infectious diseases are one of the principal bottlenecks for the European eel recovery. The aim of this study was to develop a new molecular tool to be used in host-pathogen interaction experiments in the eel. To this end, we first stimulated adult eels with different pathogen-associated molecular patterns (PAMPs), extracted RNA from the immune-related tissues and sequenced the transcriptome. We obtained more than 2x10^6^ reads that were assembled and annotated into 45,067 new descriptions with a notable representation of novel transcripts related with pathogen recognition, signal transduction and the immune response. Then, we designed a DNA-microarray that was used to analyze the early immune response against *Vibrio vulnificus*, a septicemic pathogen that uses the gills as the portal of entry into the blood, as well as the role of the main toxin of this species (RtxA1_3_) on this early interaction. The gill transcriptomic profiles obtained after bath infecting eels with the wild type strain or with a mutant deficient in *rtxA1_3_* were analyzed and compared. Results demonstrate that eels react rapidly and locally against the pathogen and that this immune-response is *rtxA1_3_*-dependent as transcripts related with cell destruction were highly up-regulated only in the gills from eels infected with the wild-type strain. Furthermore, significant differences in the immune response against the wild type and the mutant strain also suggest that host survival after *V*. *vulnificus* infection could depend on an efficient local phagocytic activity. Finally, we also found evidence of the presence of an interbranchial lymphoid tissue in European eel gills although further experiments will be necessary to identify such tissue.

## Introduction

The European eel (*Anguilla anguilla)* has received much ecological and economic attention due to current population decline. They are a commercially important species in Europe, Asia, New Zealand and the United States (FAO). Since the production cycle of the eel has not been closed under artificial conditions, eel resources are based on glass eel catch from natural stocks provoking overexploitation and overfishing [1]. In parallel many other anthropogenic factors such as climate and oceanic change, habitat loss, migration obstruction, parasite infestation, water pollution [2,3] together with several critical biological characteristics such as a migration-dependent cycle, single breeding and longevity have influenced the critical decline of the natural population over the past decades [4]. Currently, the natural stock is considered outside of safe biological limits and as a consequence the eel is listed as a critically endangered species (IUCN).

The application of transcriptomics to the biology of eels can provide a significant increase in basic information making it a powerful tool to enable basic and applied research. High-throughput sequencing technologies provide new options to characterize transcriptomes and drive the development of new molecular tools and ultimately leading to a better understanding of the biology of the species [5–12]. The current gap in knowledge concerning the biology of eels makes transcriptomics an important and attractive approach. In recent years several research groups have contributed to the significant increase of *A*. *anguilla* genomic resources that to date compromise of a draft genome for the Japanese Eel, *A japonica* [5] and the draft of the European Eel genome is available at NCBI Genomes. However eel resources for molecular studies remain scarce [11,13] particularly those addressing immunity, health and disease. Immunogenomics driven by array and RNASeq technologies has opened up new perspectives into host-pathogen interaction studies including the identification of disease-specific network signatures, candidate resistance genes and increased insight into the evolution of the immune response [9,14–16].

Infectious disease has always been a bottleneck for the management and production of fish in intensive culture systems. *Vibrio vulnificus* is one of the most devastating eel pathogens having caused the closure of several eel farms in Europe due to massive mortalities [17]. This species is subdivided into biotypes and serovars among which only biotype 2 is virulent for eels causing an haemorrhagic septicaemia known as warm water vibriosis [17–18]. The ability to infect eels relies on a virulence plasmid of around 70 Kb (pVvBt2) that encodes a toxin called RtxA1_3_ or MARTX (multifunctional, autoprocessing, repeat in toxin) type III, a fish transferrin binding protein, and several other proteins of unknown function [17–20]. RtxA1_3_ seems to be the main virulence factor responsible for eel death, as infection by immersion with a mutant defective in the toxin causes septicaemia but not death [21]. MARTX are large mosaic toxins that are secreted after contact with eukaryotic cells. The toxin forms a pore in the cell membrane and a series of internal domains with different enzymatic activities, protease activity, are liberated into the cytoplasm causing cell death [22–23]. The current hypothesis is that RtxA1_3_ interferes with eel immune cells triggering a cytokine storm responsible for the death [22].

The aim of this study was to design an immune gene-enriched oligonucleotide array for the eel and validate it by using warm water vibriosis as a disease model. To this end, we first sequenced the immune transcriptome of the eel after stimulating adult eels with different pathogen-associated molecular patterns (PAMPs), extracting RNA from the immune-related tissues and sequencing the transcriptome. Then, we designed the oligonucleotide array from this database and validated results obtained by qRT-PCR. The array was used to analyse the early immune response against *V*. *vulnificus* at the gills, the portal of entry for this pathogen into the blood [24], as well as the role of RtxA1_3_ by comparing the gill transcriptomic profiles obtained after bath challenge with the wild type strain (R99) or with a mutant deficient in *rtxA1*_*3*_ (CT285).

## Materials and Methods

### Experiments with animals

Adult eels (*Anguilla anguilla*) of about 200g (±25g) were purchased from a commercial fishery, Valenciana de acuicultura (Puçol, Valencia). Fish were maintained in our facilities in recirculating water at 15°C under a photoperiod of 12:12 (light:dark). They were acclimated to laboratory conditions for at least 15 days before experimentation. All experiments described comply with the guidelines of the European Union Council (2010/63/EU) for the use of laboratory animals and have been approved by the Department of Environment of the Generalitat de Valencia under the reference code 2014/pesca/RGP/028. Eels were divided into 4 different tanks, 5 individuals per tank. Three different experimental challenges were carried out (1 challenge/tank), which consisted of an intraperitoneal (ip) administration of 250 μl maximum volume of either; 6 mg/kg of lipopolysaccharide (LPS) from *E*. *coli* 0111:B4 (Sigma-Aldrich), 1 mg/kg of peptidoglycan (PGN) from *E*. *coli* 0111:B4 (Invivogen) or 3 mg/kg of poly I:C (Invivogen). Control individuals were injected via the intraperitoneal route with 250 μl of saline buffer. At 12 h post-treatment eels were killed by an overdose of benzocaine (Sigma-Aldrich) and liver, spleen and head-kidney were immediately dissected under sterile conditions and frozen in liquid nitrogen.

### Library construction and sequencing

RNA extraction of individual tissues was performed using the commercial kit RNeasy MIDI kit (Quiagen), following the manufacturer’s instructions. RNA concentration and purity were measured using a NanoDrop1000 (Thermo scientific). RNA integrity was checked with an Agilent Bioanalyzer 2100 using the RNA nano chip kit (Agilent technologies). Four individuals from each group were used leading to a total of 48 samples. Samples with a RNA integrity number (RIN) value > 7.5 were pooled for cDNA library construction and sequencing.

Full-length-enriched double stranded cDNA was synthesized from 1.5 μg of pooled total RNA using MINT cDNA synthesis kit (Evrogen) according to manufacturer's protocol, and was subsequently purified using the QIAquick PCR Purification Kit (Qiagen). The amplified cDNA was normalized using Trimmer kit (Evrogen) to minimize differences in representation of transcripts. The method involves denaturation-reassociation of cDNA, followed by a digestion with a Duplex-Specific Nuclease (DSN) [25–26]. The enzymatic degradation occurs primarily on the highly abundant cDNA fraction. The single-stranded cDNA fraction was then amplified twice by sequential PCR reactions according to the manufacturer's protocol. Normalized cDNA was purified using the QIAquick PCR Purification Kit (Qiagen).

Five hundred nanograms (500ng) of normalized cDNA were used to generate the 454 libraries. cDNA was fractionated into small, 300- to 800-basepair fragments and specific A and B adaptors were ligated to both the 3' and 5' ends of the fragments. The A and B adaptors were used for purification, amplification, and sequencing steps. Two cDNA library replicates were obtained and two runs of each library were performed on the GS-FLX using Titanium chemistry, obtaining 4 independent raw data files GN8XUQJ01, GN8XUQJ02, GPBPVW401 and GPBPVW402. All reagents and protocols used were from Roche 454 Life Sciences, USA. RNA was normalized, processed and sequenced at the Unitat de Genòmica (CCiT-UB, Barcelona, Spain). The sequences have been deposited in the NCBI Short Read Archive (SRA) under accession SRA090946 (Experiment SRX304888). Only high quality reads passing filtering were used for further assembly using Newbler version 2.3 program (454 Life Science-Roche) with the parameters set to default. This Transcriptome Shotgun Assembly project has been deposited at DDBJ/EMBL/GenBank under the accession GBXM00000000. The version described in this paper is the first version, GBXM01000000.

Assembled contigs and singletons were annotated searching sequence homologies against NCBI’s non-redundant protein (nr) and NCBI's redundant nucleotide database (nt) by bestBLAST iterative methodology. Script consisted of four step iterative blast, combining BLASTX ➔ BLASTN ➔ BLASTX ➔ BLASTN, until a best hit description was assigned. The two first rounds were based on best description and the two next rounds based on e-values. If no description was found after the 4 rounds, sequences appear as “no hit found”. The e-value cut-off was set to 1E^-05^ and the bestBLAST hit with highest similarity and lowest e-value was assigned as the mRNA transcript identity. Hit descriptions were also filtered in order to remove uninformative identities.

### Custom microarray design

The eel-specific gene expression microarray (4x44K) slides were custom designed with the eArray software (Agilent technologies), following MIAME guidelines [27]. A custom selection of transcripts from the *de novo* annotation, enhancing immune-related transcripts, was used for probe design. The arrays contained in total 41,383 probes of 60-oligonucleotide length. These probes were distributed as the following, 11,096 annotated singlets were chosen and two probes per target were added (22,192), a total of 6,397 annotated contigs with 3 probes per target (19,191) and the rest were filled with internal control probes. Settings used were based on the following: base composition methodology, best probe methodology, and designed with a 3’ bias.

### Bacterial strains and growth conditions

*V*. *vulnificus* strains used in this study were the wild type strain (CECT4999 or R99) and an *rtxA1*_*3*_ double deletion strain derived from R99 that is non-virulent (CT285) [19]. *V*. *vulnificus* strains were grown on Tryptone Soy Agar (TSA) or Broth (TSB) supplemented with 0.5% (w/v) NaCl (TSB-1 or TSA-1) medium at 28°C for 24 hours to reach a concentration of 10^9^ colony forming units (CFU)/ml for bath infection. Bacterial concentration was checked before and after bath infection in TSA-1 plates.

### *In vivo* bacterial challenge

Farmed European eel (*A*. *anguilla*) of approximately 100 gr (±15 gr) were purchased by a local eel-farm. Fish were placed in quarantine in 170 L-tanks (6 fish per tank) containing brackish water (1.5% NaCl, pH 7.6) with aeration, filtration and feeding systems at 25°C for one week. Fish were distributed into two groups of 12 individuals plus control (C) and handling control (HC) groups with an n = 4 each. Individuals were infected by bath immersion with R99 or CT285 at a dose of 1x10^7^ CFU/ml (Lethal dose 50% previously determined for R99 infection for this eel stock). After 1 h of immersion, fish were transferred into new tanks, containing clean water (1.5% salt, at 20°C) and kept under constant conditions until sampling. Fishes were randomly sacrificed at 0, 3 and 12 h post-infection in groups of 4 individuals with an overdose of benzocaine (Sigma-Aldrich) and gills were quickly dissected and treated with RNAlater and stored at -80°C until use.

Control group were non-previously manipulated fishes and handling control group were fishes that had been manipulated as the challenged with clean-fresh water. All experiments described comply with the guidelines of the European Union Council (2010/63/EU) for the use of laboratory animals and have been approved by the department of environment of the Generalitat de Valencia under the reference code 2014/pesca/RGP/028.

### Microarray protocol

Gills were shredded with a Polytron homogenizer (PT 1600E) by adding 1 ml of TRI Reagent (Sigma-Aldrich) and total RNA was purified following manufacturer’s instructions. Possible contaminating DNA was eliminated using RNeasy MinElute Cleanup kit (Qiagen) with a DNase I (Qiagen) digestion at room temperature for 15 min according to manufacturer’s protocol. RNA integrity and quality were verified with an Agilent 2100 Bioanalyzer using the RNA 6000 Nano Chip kit. High quality samples, with an RNA Integrity Number (RIN) over 7.5 were obtained and used for microarray analysis. Sample quantifications were check by a Nanodrop ND-1000 (Thermo Scientific). A total of 200 ng of RNA from each sample was used for indirect labelling with Cy3 Dye (Agilent), labelled cRNA was purified using RNeasy mini spin columns (Qiagen) and quantified. After yields and specific activity was checked, hybridization was performed at 65°C for 17h, employing 1.65 μg of cyannine 3-labeled cRNA of each sample to hybridize into custom eel-specific microarray (ID 042990, Agilent) using Agilent’s GE Hybridization kit. All procedures were performed following manufacturer’s instructions for one-color microarray-based gene expression analysis along with Agilent’s one-color RNA spikeIn kit. Oligonucleotide microarrays slides were scanned with Agilent Technologies Scanner, model G2505B.

Microarray data were extracted from raw data image with Feature extraction software (Agilent technologies). Quality reports were generated and checked for each array. Extracted raw data were imported and analyzed with Genespring 12.5 GX software (Agilent technologies). The 75% percentile normalization was used to standardize arrays for comparisons. All samples were analyzed at gene-level by two different analytical approaches, by loop analysis and relative analysis against a reference sample. Principal component analysis (PCA) was used as a quality control, to detect any outlier sample, and to describe differences between groups. Statistical analysis available in Genespring software were run, one-way ANOVA (p<0.05) followed by Tukey’s pairwise comparisons were selected to describe transcriptomic profile differences along the time for each strain and between strains.

The complete design has been summited to Gene Expression Omnibus (GEO) database with the number GSE45163 linked to reference platform GPL16775.

### Microarray validation by absolute quantification

Specific primers for *mrlc2*, *clec1*, *casp1*, *casp3*, *mx*, *il1r2*, *cxcr4* and *pcna* transcripts were designed and analyzed (S1 File). Plasmids were obtained using the p-GEM easy vector (Promega) and transformed into JM109 competent cells (Promega) to be used for sequencing and standard curve generation. 400 ng of total RNA of all samples included in the microarray analysis were used to synthesize cDNA with SuperScript III Transcriptase (Invitrogen) and OligodT(Promega). cDNA was used as a template for absolute quantification in real-time RT-PCR (qRT-PCR) expression analysis. The copy number of each transcript was analysed using the MyIQ real-time PCR system (Bio-Rad, CA). Standard curves (Ct-Threshold cycle versus log copy number) of each transcript were done with serial dilutions of DNA plasmid purifications from 10^2^ to 10^8^ copies. Each sample was tested in triplicate in a 96-well plate (Bio-Rad, CA). The reaction mix (15 μL final volume) consisted of 7.5 μL of iQ SYBR Green supermix (Bio-Rad), 0.75 μL of each primer (500 nM final concentration), 2.5 μL of H_2_O, and 3.75 μL of a 1/10 dilution of the cDNA sample. The thermocycling program consisted of one hold at 95°C for 4 min, followed by 40 cycles of 10 s at 95°C and 45 s at 60°C. Data were analyzed by one-way analysis of variance (ANOVA) followed by the post hoc multiple comparison by Bonferroni’s method that was run for each gene to determine differences between groups (p<0.05).

## Results

### 454 Transcriptome assembly and data analysis

We obtained a total of 2.213,260 high-quality reads from 2 sequencing runs of the two library replicates. Reads were trimmed and *de novo* assembled using Newbler v2.3. During the assembly, 2.059,281 reads were incorporated into 32,687 contiguous sequences (contigs). 153,979 reads could not be matched against any other (no assembly) and remained as singlets, resulting in a total of 186,666 putative European eel transcripts of >50 base pairs. The average length of the contigs was 576 base pairs, with more than 14000 contigs having a length >500 base pairs, the average number of reads within the library per contig was 67 (median = 19) (Table 1). The sequences were deposited in Genbank with accession numbers GBXM00000001 to GBXM00024456.

**Table 1:** 454 reads statistics. Description of different properties of sequenced transcripts, assembly and annotation.

454 Statistics		
**Singlets**		
	**Total**	**153979**
	**with annotation**	**32171 (20.9%)**
	max. length	574
	min. length	50
	average length (bp)	257
**Contigs**		
	**Assembled**	**32687**
	average length (bp)	576
	average reads per contig	63
	**with annotation**	**12896 (39.5%)**
	max. length	7838
	min. length	50
	average length (bp)	724
**Total number of reads**		**> 2 x 10** ^**6**^

Annotation of all sequences was carried out with a four-step script combining BLASTN/BLASTX algorithms, which prioritizes hits with reference to other fish species in the two first rounds and the next two are based on best e-value match. An e-value cut-off of 10^−5^ was set and sequences with a length <50 base pairs (bp) were removed from the annotation. This resulted in a total of 109227 sequences with an estimated redundancy of 8.3% (95% sequence identity; CDHIT [28]. The annotation and therefore assignation of a bestBLAST-hit, for 39.5% (12,896 sequences) of the contigs and for 20.79% (32,171 sequences) of the singlets led to a total of 45,067 new descriptions. For contigs, the longest consensus sequence with bestBLAST hit assigned was 7,838 bp. The annotation ratio was directly correlated to transcript length, as longer sequences have an increased probability to identify an informative BLAST hit match. The percentage of annotation for transcripts with > 500 base pairs was above 45%. Our annotation resulted in a total of 12896 mRNA contigs of which around 8% were identified with respect to known immune-relevant transcripts in other species including, immune system recognition, signalling transduction and response molecules (Table 2). These transcripts include significant number of putative toll-like receptors (*tlr1*, *2*, *3*, *5*, *5s*, *13*, *20*, *21*) and representation from other receptor groups including immunoglobulin, B-cell, T-cell, peptidoglycan, complement, NOD-like, mannose and cytokine and chemokine receptors (CC and CXC receptors). Important elements of the immune response were found such as, inflammatory cytokines and chemokines (*ccl4*, *il8*, *il6*, *il1b*, *ifna*, *infg*), complement system components (*c3*, *c4*, *c6*, *cfh*), immunoglobulins (*IgM*, *IgT*) and several immune-related membrane proteins and other molecules (*cd81*, *cd83*, B-cell ligands, *hsps*, *mhcI*, *mhcII*, *transferrin*, *lyzozyme*). In addition to recognition and immune response mRNAs, other transcripts involved to immune-signalling pathways and adapters were annotated as well as multiple adapter molecules and transcription factors such as, *nfkb*, *myd88*, *stat6*, *tollip*, *jnk*, *traf*, *trif* and some *mapk*. This provides a powerful tool for the study of the underlying molecular mechanisms of the eel immune system against infections.

**Table 2:** Summary of selected immune-relevant transcripts identified.

Name	Description	E-value	Best BLAST hit
**Pattern recognition genes**			
CR	Complement receptor	8.00E-26	*Oncorhynchus mykiss*
CtlR	C-type lectin receptor	9.00E-64	*Salmo salar*
MR	Mannose receptor C1-like protein	1.00E-171	*Danio rerio*
MR	Mannose-6-phosphate receptor	7.00E-25	*Salmo salar*
NLR	NOD-like receptor	2.00E-27	*Ictalurus punctatus*
SR	Scavanger receptor	2.00E-18	*Strongylocentrotus purpuratus*
TLR1	Toll-like receptor 1	5.00E-18	*Takifugu rubripes*
TLR13	Toll-like receptor 13	2.00E-49	*Salmo salar*
TLR2	Toll-like receptor 2	1.00E-158	*Cyprinus carpio*
TLR3	Toll-like receptor 3	6.00E-46	*Paralichthys olivaceus*
TLR5	Toll-like receptor 5	1.00E-17	*Ictalurus punctatus*
TLR5s	Toll-like receptor 5 soluble	4.00E-09	*Takifugu rubripes*
**Cytokine & chemokine receptors**			
CC receptor	C-C chemokine receptor type 4	9.00E-54	*Danio rerio*
CXC receptor	C-X-C chemokine receptor 4a	1.00E-139	*Rattus norvegicus*
IL-10R	Interleukin-10 receptor	3.00E-50	*Salmo salar*
IL-1R	Interleukin-1 receptor	2.00E-33	*Salmo salar*
IL-2R	Interleukin-2 receptor	5.00E-22	*Danio rerio*
IL-6R	Interleukin-6 receptor	9.00E-20	*Salmo salar*
TNF decoy receptor	TNF decoy receptor	7.00E-88	*Conger myriaster*
TNFR	Tumor necrosis factor receptor	9.00E-171	*Xenopus laevis*
**Immunoglobulin receptors & others**			
IGγR	Immunoglobulin gamma receptor	5.00E-10	*Salmo salar*
IgεR	Immunoglobulin epsilon receptor	6.00E-18	*Esox lucius*
LYSMD2	LysM and peptidoglycan-binding domain 2	7.00E-60	*Osmerus mordax*
PIR	Polymeric immunoglobulin receptor	6.00E-10	*Paralichthys olivaceus*
PTX	Pentraxin	2.00E-45	*Cyprinus carpio*
TCRα	T-cell receptor alpha	2.00E-101	*Ictalurus punctatus*
TCRβ	T-cell receptor beta	1.00E-22	*Oncorhynchus mykiss*
TCRγ	T-cell receptor gamma	2.00E-22	*Ictalurus punctatus*
TfR	Transferrin receptor	1.00E-41	*Danio rerio*
**Inflamatory cytokines & chemokines**			
CCL4	CC chemokine ligand 4	2.00E-18	*Oncorhynchus mykiss*
CK3	CC chemokine CK3	8.00E-20	*Sparus aurata*
CXCL13	CXC chemokine ligand 13	3.00E-12	*Salmo salar*
IFNα	Interferon alpha	7.00E-25	*Salmo salar*
IFNγ	Interferon gamma	5.00E-87	*Anoplopoma fimbria*
IL-10	Interleukin-10	7.00E-37	*Oncorhynchus mykiss*
IL-11	Interleukin-11	1.00E-30	*Cyprinus carpio*
IL-16	Interleukin-16	9.00E-19	*Oncorhynchus mykiss*
IL-18	Interleukin-18	2.00E-26	*Oncorhynchus mykiss*
IL-1β	Interleukin-1 beta	5.00E-20	*Conger myriaster*
IL-8	Interleukin-8	7.00E-32	*Labeo rohita*
IL-6	Interleukin-6	5.00E-15	*Oncorhynchus mykiss*
SCYA112	CC chemokine SCYA112	3.00E-24	*Ictalurus punctatus*
TNFα-IP2	Tumor necrosis factor alpha-induced protein 2	7.00E-19	*Salmo salar*
TNFα-IP8	Tumor necrosis factor alpha-induced protein 8	2.00E-80	*Danio rerio*
**Complement system**			
C3	Complement component C3	3.00E-127	*Oncorhynchus mykiss*
C4	Complement component C4	7.00E-178	*Oncorhynchus mykiss*
C6	Complement component C6	7.00E-58	*Danio rerio*
C9	Complement component C9	1.00E-164	*Oncorhynchus mykiss*
CFH	Complement factor H	2.00E-44	*Danio rerio*
**Immunoglobulins & other immune-related molecules**			
A2M-1	alpha-2-macroglobulin-1	6.00E-151	*Cyprinus carpio*
Bcl2	B-cell ligand 2	6.00E-25	*Danio rerio*
Bcl6	B-cell ligand 6	2.00E-29	*Danio rerio*
CD81	CD81 antigen	1.00E-84	*Ictalurus punctatus*
CD83	CD83 antigen	2.00E-35	*Oncorhynchus mykiss*
HSP70	Heat shock protein 70	0.0	*Hypophthalmichthys molitrix*
HSP90	Heat shock protein 90	0.0	*Paralichthys olivaceus*
IgG	Immunoglobulin gamma light chain	2.00E-12	*Ictalurus punctatus*
IgL	Immunoglobulin lambda light chain	5.00E-31	*Larimichtys crocea*
IgM	Immunoglobulin mu heavy chain	3.00E-46	*Oncorhynchus mykiss*
IgT	Immunoglobulin tau heavy chain	1.00E-05	*Oncorhynchus mykiss*
Lyzozyme	Lyzozyme	5.00E-78	*Oncorhynchus mykiss*
MHCI	Major histocompatibility complex class I	3.00E-76	*Danio rerio*
MHCII	Major histocompatibility complex class II	1.00E-42	*Epinephelus akaara*
SART1	T-cell-recognized antigen	2.00E-124	*Tetraodon nigroviridis*
TCF7	T-cell transcription factor 7	3.00E-65	*Homo sapiens*
TF	Transferrin	2.00E-103	*Salmo trutta*
TF2	Transferrin 2	4.00E-14	*Salmo trutta*
**Adapters and signal transducers**			
IRAK4	Interleukin-1 receptor-associated kinase 4	8.00E-22	*Coregonus maraena*
IRF2	Interferon regulatory factor 2	3.00E-50	*Salmo salar*
IRF3	Interferon regulatory factor 3	4.00E-41	*Salmo salar*
IRF9	Interferon regulatory factor 9	2.00E-17	*Salmo salar*
JAK3	Janus kinase 3	8.00E-135	*Siniperca chuatsi*
JNK	c-Jun N-terminal kinase	4.00E-80	*Ctenopharyngodon idella*
MAPK1	Mitogen-activator protein kinase	7.00E-61	*Ictalurus punctatus*
MAPK2	Mitogen-activator protein kinase	1.00E-71	*Danio rerio*
MyD88	Myeloid differentiation primary response gene 88	1.00E-14	*Plecoglossus altivelis*
NFκB	Nuclear factor-kappa-B-activating protein	6.00E-65	*Danio rerio*
STAT6	Signal transducer and activator of transcription 6	7.00E-85	*Tetraodon fluviatilis*
TOLLIP	Toll-interacting protein	2.00E-16	*Salmo salar*
TRAF3	TNF receptor-associated factor 3	1.00E-27	*Xenopus tropicalis*

### Transcriptional regulation in gills

**Fig 1:**
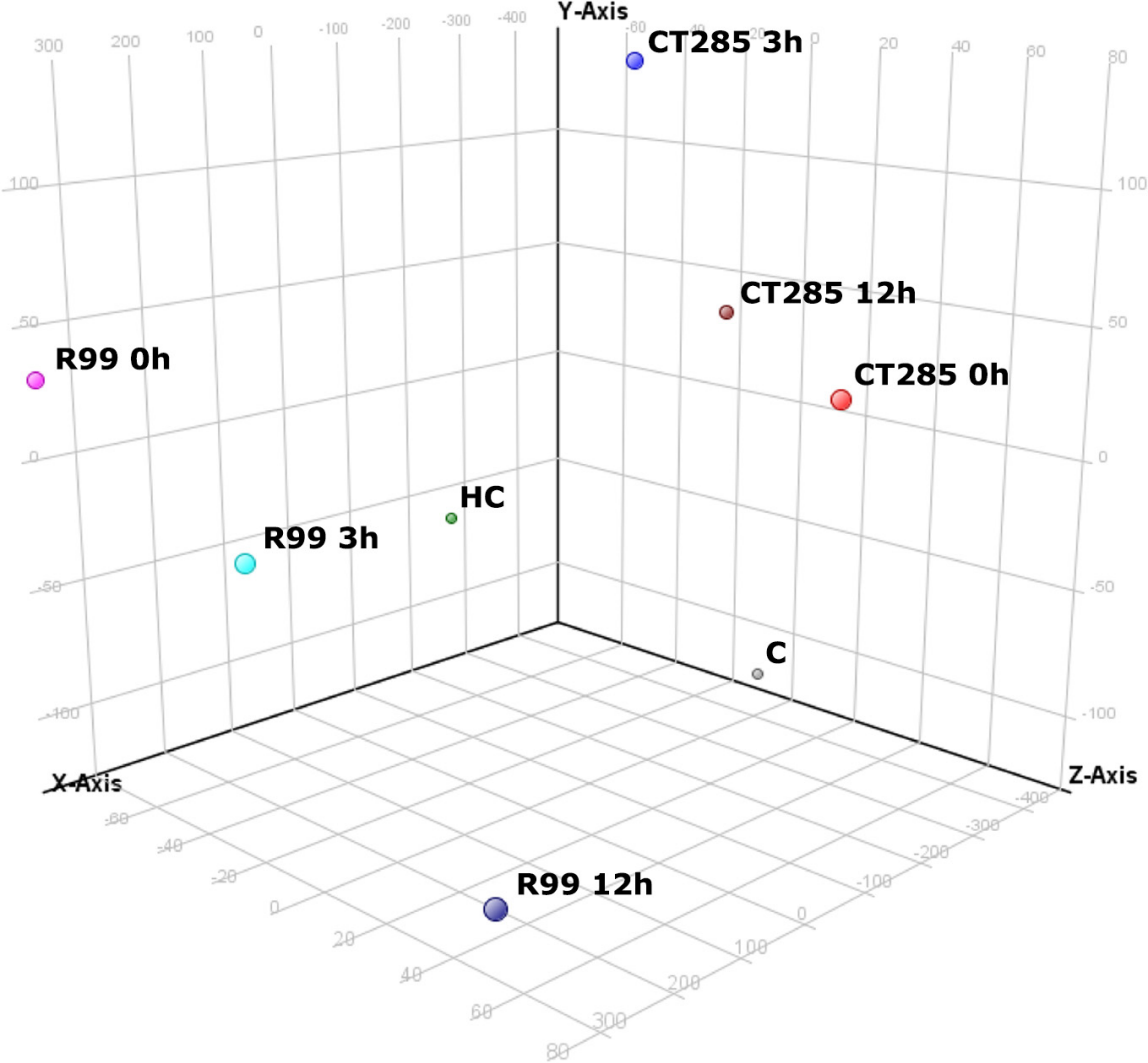
Principal Component Analysis (PCA) 3-D plot of the gills samples grouped by challenge. Three principal components are represented, PC1 on X-axis (54.78%), PC2 on Y-axis (14.53%) and PC3 on Z-axis (9.14%).

In order to characterize the response of adult European eels to *V*. *vulnificus* Bt2 SerE infection and the role of RtxA1_3_ in the early host-pathogen interaction, we performed a time-dependent transcriptome analysis of adult eels bath-infected with either, R99 or a double mutant defective in RtxA1_3_ production (CT285). This experimental design allowed us to interpret the specific response in the gills against *V*. *vulnificus* as well as gain insight into the role of the RtxA1_3_ toxin during the early steps of warm water vibriosis. Gill samples were successfully hybridized onto the arrays. After data normalization and removal of outliers and flags, data was grouped by challenge (n = 3 per challenge). Microarray analysis was performed at gene-level (p<0.05) using GeneSpring. Principal component analysis (PCA) was used as a quality control for the samples and also as a simplified methodology for visualization of the data sets [29]. The PCA divided into 3 principal components that explain 78.5% of the total variance revealing a clear differential response between wild type and mutant strains. This allowed us to group all samples into 3 well-defined clusters corresponding to wild type, mutant strain infection and control groups (Fig 1).

**Fig 2:**
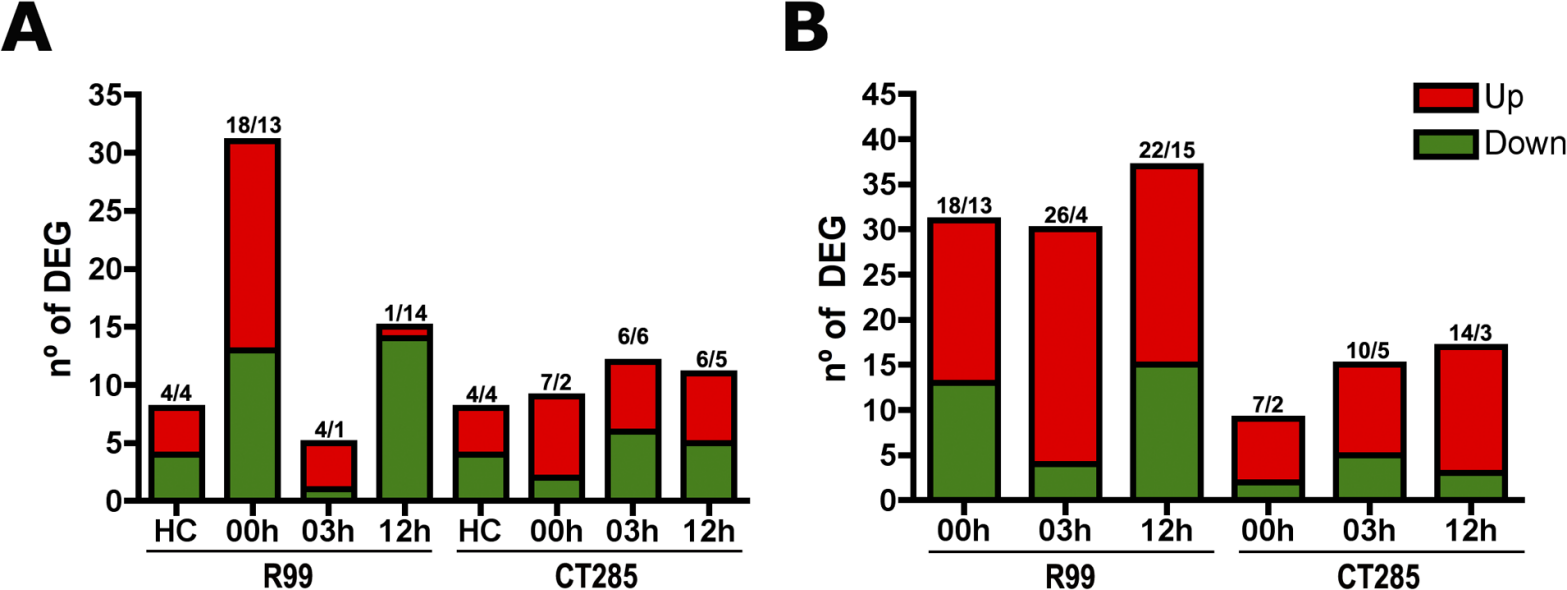
Magnitude of response by mean representation of differential expressed genes (DEGs). Bars represent the sum of upregulated (in red) and downregulated (in green) DEGs of each sampling group (n = 3). Numbers on the top of each bar represent DEGs number up/downregulated. **(A)** Loop analysis approach and on **(B)** Relative analysis against handling control group (HC). Numbers above the columns indicate upregulated/downregulated DEGs.

**Fig 3:**
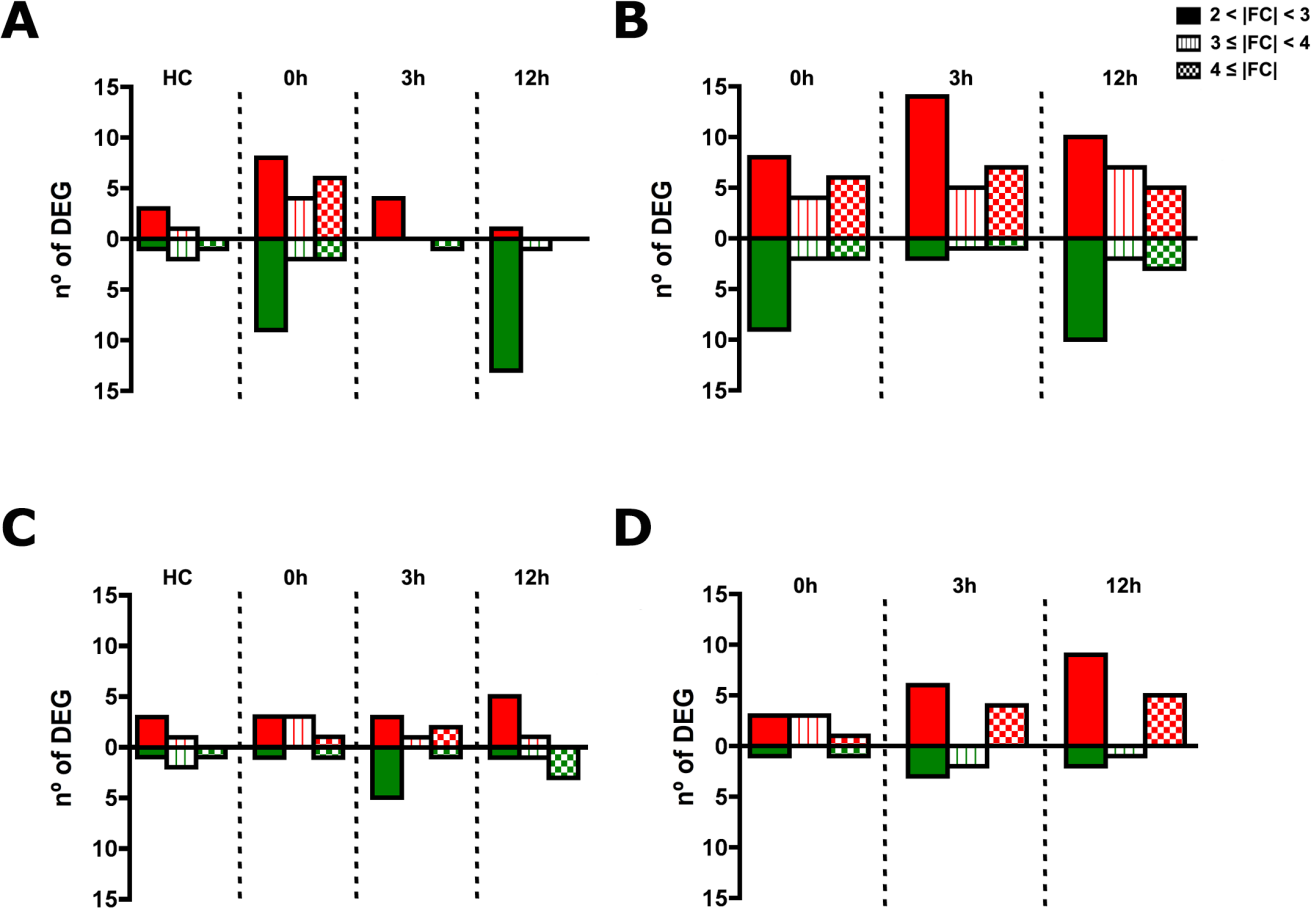
Intensity of response after R99 infection (A-B) or CT285 infection (C-D) represented in number on DEGs grouped in 3 groups by fold change (FC), from 2 to 3, from 3 to 4 and over 4. Red bars are upregulated transcripts and green bars are downregulated transcripts. **(A and C)** Loop analysis approach and on **(B and D)** Relative analysis against handling control group (HC).

Microarrays were analyzed following minimum information about microarray experiment (MIAME) guidelines manual [27]. Two different analytical approaches were used for the microarray data to provide the most complete interpretation of the results. On one hand, we performed a loop analysis [30] (Table A in S2 File), revealing the modulation of the response in a time-dependent manner and thus assessing transient changes (Figs 2A, 3A and 3C). On the other hand, we carried out a relative analysis, comparing all groups to the handling control (HC) group, used as a reference (Table B and Fig A-B in S2 File). This evaluated accumulated changes against the starting point (Figs 2B, 3B and 3D).

Our results based on the number of differentially expressed genes (DEGs; p<0.05) with a fold change (FC) of >2 obtained from both analytical approaches and for both infections with *V*. *vulnificus*, identified 31 DEGs for R99-infected eel samples after 1 h of bath infection (time 0) and 12 for CT285 infection at 3 h post-challenge by loop analysis. With relative analysis we were able to identify 37 and 17 DEGs for R99 and CT285 strains respectively both corresponding to 12 h post-infection (Fig 2). Thus maximum change (FC) in intensity for the measured mRNAs occurred in the gills during infection with the wild type strain (R99). This corresponded to the largest change in the transcriptome registered throughout the experiment. In the same experimental group (R99) 5 and 15 DEGs observed at 3 and 12 h post-infection respectively. In the CT285 infection group the magnitude of the changes in the transcriptome observed were constant (mean = 10; SD = 1.8) throughout the experiment with 9, 12, and 11 DEGs at 0, 3 and 12 h post-infection observed respectively thus showing no time-dependent response to infection in respect to the number of transcripts (Fig 2A). A total of 8 DEGs were modulated between the control and handling control group (Fig 2A), which may be due to the stress provoked by handling or other unknown environment stimuli [31]. Therefore, the handling control group was used as a reference group for further evaluation of the results by the relative analysis approach.

**Fig 4:**
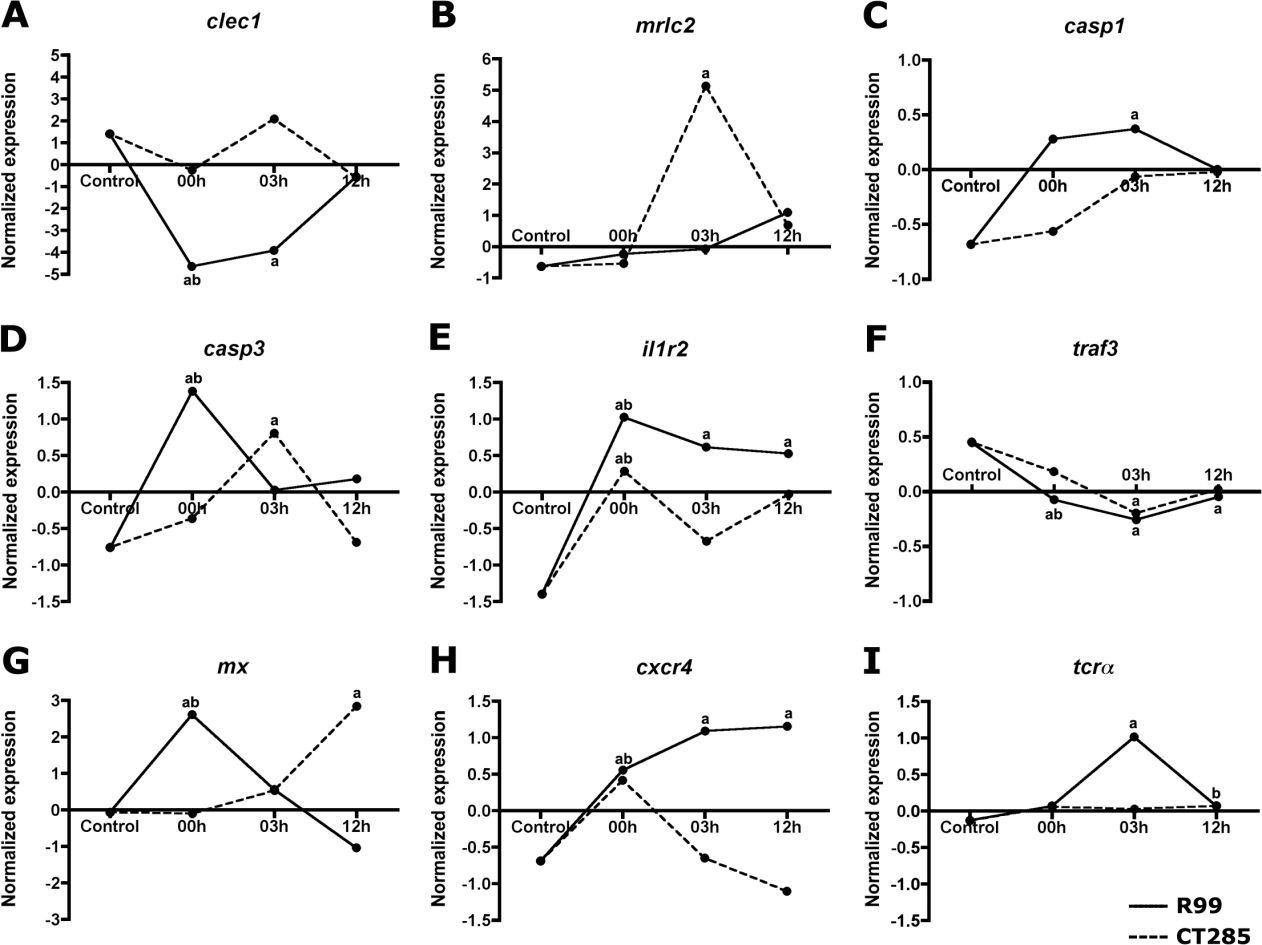
Detailed representation of most relevant up and downregulated transcripts by mean normalized array expression, corresponding to (A) C-type lectin 1 (*clec1*), (B) to Myosin regulatory light chain 2 (*mrlc2*), (C) to Caspase-1 (*casp1*), (D) to Caspase-3 (*casp3*), (E) to Interleukin 1 receptor type 2 (*il1r2*), (F) to TNF receptor-associated factor 3 adaptor (*traf3*), (G) to Myxovirus resistance (*mx*), (H) to CXC chemokine receptor type 4 (*cxcr4*) and (I) to T-cell receptor V alpha (*tcrα*). Continuous line represents R99 infection and discontinuous line the CT285 infection. *a* represents statistical significance (p<0.05) relative to HC; and *b* statistic significance of p<0.05 by loop analysis.

Results obtained by relative analysis revealed that the magnitude of activation of the transcriptome remains constant until 12 h post-infection, with mean DEGs of 32.7 (SD = 3.8) for R99 and 13.7 (SD = 4.2) for CT285 (Fig 2B). Therefore the results of both analyses indicate that the timing of gill transcriptome activation to the infection is very short (Fig 2A). This short time period where the gill tissue contacts with the bacteria prior to secondary colonization into the individual may explain the relatively weak activation of differential mRNA expression and the low intensities (FC 2–3) reported suggesting that the gills do not strongly respond to *V*. *vulnificus* colonization (Fig 3). However some specific mRNAs with higher intensities were also identified including the *C-type lectin 1* (*clec1*) and *myosin regulatory light chain* (*mrlc2*). *Clec1* transcripts were the most strongly regulated by R99 bacteria, with 65.69 fold downregulation after bath (1 h) and 39-fold down after 3 h post infection, this effect is lost at 12 h as clec1 mRNA levels recovered basal levels (Fig 4A). Myosin regulatory light chain 2 (*mrlc2*) was the most regulated mRNA transcript by CT285, 54-fold upregulated, after 3 h of infection with the mutant strain, whereas in wild type infected eels it remained unchanged until 12 h post-infection (Fig 4B). Further classification of DEGs obtained by both approaches using the GeneCards database [32] highlighted that majority of DEGs are involved in the immune response. The most relevant immune-related regulated mRNA transcripts are shown in Table 3 for R99 infection and Table 4 for CT285.

**Table 3:** List of relevant immune-related transcripts regulated during *V*. *vulnificus* R99 infection by loop analysis. Asterisk (*) represents relevant mRNAs with FC<2, however with statistically significant difference (p<0.05).

**RELEVANT mRNAs REGULATED by R99 strain**			
**At 00h post-infection**			
**Description**	**Regulation**	**Fold change**	**Persistence**
**MX**	Up	6.45	-
**IL1R type II**	Up	5.37	Until 12h
**Caspase-3**	Up	4.40	-
**CXCR4**	Up	2.38	Until 12h
**Reactive oxigen species modulator 1**	Down	-2.35	-
**60S ribosomal protein L4-A**	Down	-2.66	-
**Heat shock 70kDa protein 8**	Down	-2.69	Until 12h
**C-type lectin 1**	Down	-65.69	Until 03h
**Ubiquitin-conjugating enzime E2L 3**	Up	1.85 (*)	Until 12h
**TRAF3**	Down	-1.43 (*)	Until 12h
** **	** **	** **	** **
**At 12h post-infection**			
**Description**	**Regulation**	**Fold change**	**Persistence**
**Transcription factor 12 (TCF12)**	Down	-2.07	-
**T-cell receptor alpha variable**	Down	-2.12	-

**Table 4:** List of relevant immune-related mRNAs regulated during *V*. *vulnificus* CT285 infection by loop analysis. Asterisk (*) represents relevant mRNAs with FC<2, however with statistically significant difference (p<0.05).

**RELEVANT mRNAs REGULATED by CT285 strain**			
**At 00h post-infection**			
**Description**	**Regulation**	**Fold change**	**Persistence**
**IL1R type II**	Up	3.23	-
** **			
**At 03h post-infection**			
**Description**	**Regulation**	**Fold change**	**Persistence**
**Myosin regulatory light chain 2**	Up	50.88	-
**Proteasome beta type-9**	Up	4.34	-
** **			
**At 12h post-infection**			
**Description**	**Regulation**	**Fold change**	**Persistence**
**Nitric oxide synthase adaptor protein c**	Up	2.15	-
**Nuclear factor interleukin-3-regulated protein**	Up	2.62	-

Globally the wild type strain, Rtx-dependent, induced stronger transcriptomic changes than the double mutant although multiple regulated mRNAs could also be attributed to the general host immune response against *V*. *vulnificus*. In a RTX-dependent manner both CXC chemokine receptor type 4 (*cxcr4*) and the T-cell receptor V alpha (*tcr* **α**) were rapidly upregulated: *cxcr4* with a 2.38-fold at time 0 with a maximum of 3.59-fold maximum at 12 h (Fig 4H) and (*tcr* **α**) with a 2.2-fold increase at 3 h (Fig 4I). Both *caspase-1* (*casp1*) and *caspase-3* (*casp3)* were also differentially regulated in a strain specific manner. Interestingly, *casp1* was exclusively upregulated by R99 with a statistically significant 2-fold increase 3 h post-infection (Fig 4C) whereas *casp3* was regulated by both strains, R99 with a 4.4-fold increase and CT285 with a 3-fold increase at different time points (Fig 4D). We also identified Rtx-independent mRNAs (found in both challenges) including interleukin 1 receptor II (*il1r2*) that was significantly more upregulated in the gills from R99-infected eels (Fig 4E) and TNF receptor-associated factor (*traf3*) that exhibited similar behaviour during both infections (Fig 4E and 4F). The Myxovirus resistance mRNA (*mx)* was also regulated by both infections although with a different temporal pattern where it was rapidly upregulated (6.45 fold) by R99 bath infection whereas a delayed response (12 h) was observed with a similar intensity (8-fold) in CT285 bath infection (Fig 4G) suggesting that different cellular activation pathways may be utilized.

Validation of the microarray results was performed by absolute quantification RT-qPCR analyzing 8 different transcripts, *mrlc2*, *clec1*, *casp1*, *casp3*, *mx*, *il1r2*, *cxcr4* and *pcna*. Individual fold-change regulation values are summarized in Table 5, supporting and corroborating the results obtained in our transcriptomic analyses.

**Table 5:** Microarray validation analysis by RT-qPCR absolute quantification based on mRNA fold-change.

					
		Microarray		RT-qPCR	
Description	Challenge	Fold Change	Regulation	Fold Change	Regulation
***casp1***	R99 03h *vs* HC	+2.07	Up	+174.87	Up
***casp3***	R99 00h *vs* HC	+4.40	Up	+10.00	Up
***clec1***	R99 00h *vs* HC	-65.69	Down	-530.69	Down
	R99 03h *vs* HC	-39.41	Down	-26.49	Down
***cxcr4***	R99 00h *vs* HC	+2.38	Up	+5.74	Up
***il1r2***	R99 00h *vs* HC	+5.37	Up	+3.25	Up
***mrlc2***	CT285 03h *vs* HC	+54.06	Up	+50.75	Up
***mx***	R99 00h *vs* HC	+6.45	Up	+7.41	Up
***pcna***	R99 00h *vs* HC	+3.48	Up	+8.07	Up

## Discussion

### Immune-enriched transcriptome sequencing

Two parallel target cDNA libraries were generated from total RNA in tissue pools; liver, spleen and head kidney obtained from individual fish challenged with one of the different PAMPs selected (LPS, PGN and Poly I:C) and a control group. This led to the production of > 2 x 10^6^ high quality reads. cDNA libraries of liver, spleen and kidney have been proven to be an excellent source of transcript information concerning immune function in fish due to their central role in response to infectious diseases [33–36]. Kidney, spleen and thymus are considered the major lymphoid organs in teleosts [37–39] and the liver has an important role as an immune organ producing a significant number of antimicrobial peptides [38]. Furthermore, experimental activation of immune responses can be conducted by stimulation with different PAMPs, widely used in research to mimic viral (poly (I:C)), bacteria (LPS, PGN, CpGs) and fungal (Zymosan) infections [39]. The use of PAMPs as a replacement for a real pathogenic infection facilitates reproducibility of the experiments, design and data analysis by avoiding traces of pathogen genomic material and increased experimental variation.

*A*. *anguilla* occupies a basal position in the teleost phylogenetic tree and also is phylogenetically distant from other well described species [40–42]. Despite this potential limitation a significant number of novel descriptions, 40% for contigs, mainly annotated in *O*. *mykiss* and *D*. *rerio* transcripts (Table 2) were obtained. This was similar to a previously reported study in eel larvae that achieved 36% of contig *de novo* annotation [43]. Interestingly sequence annotation success was similar to other 454 *de novo* sequencing projects targeting teleost species with better transcriptomic resources such as the turbot with a 45% of annotated contigs with equivalent e-value cut-off [43–45]. However it was significantly less than annotations obtained from well-resourced teleost species such as common carp with a 52%, or sea bream larvae and juveniles with 66% and 51% respectively [43,45]. The obtention of 8% of immune-related transcripts (978 annotated transcripts) highlights the success of our immune-enriched library by PAMP stimulation with a similar performance when compared to other studies such as the 9.5% (2,241 transcripts) obtained in turbot infected with *E*. *scophtalmi* [44]. In context this is significantly higher than the 1.27% and the 1.35% reported from sequencing in non immune-enriched libraries from *S*. *aurata* larvae and juveniles respectively [43,45]. From these resources we then designed an eel custom microarray platform with enhanced representation of immune-related transcripts to study a broad range of diseases, both bacterial and viral.

### Application of the eel-microarray to disentangle the early steps of warm water vibriosis

We selected warm water vibriosis caused by *V*. *vulnificus* Bt2 SerE as the disease model to test our custom array. Warm water vibriosis is one of the most devastating diseases affecting farmed eels grown in brackish water [17]. The disease is transmitted through water where the pathogen colonizes the gills and spreads to blood and internal organs causing death by primary sepsis [24]. This pathogen produces a toxin, called RtxA1_3,_ that has been proposed as the main virulence factor responsible for eel death [22]. Our array would constitute an ideal tool to test the veracity of this hypothesis by comparing the eel immune response against the pathogen and against a mutant deficient in the toxin. In this study, we have used both strains to infect eels through water and analyzed the differential immune response in the portal of entry, the gills, from 0 to 12 h post-infection.

Principal component analysis, as well as the magnitude of response much higher in R99-infected than CT285-infected eels, demonstrates that host response is directly or indirectly related to RtxA1_3_ production, as the absence of the toxin in the bacteria provokes completely different host transcriptomic response profiles. Although DEGs numbers were not very high, with a maximum of 37 for R99 and 17 for CT285, a high percentile were related to the immune system suggesting that gills, as a portal of entry, may have an important role in immune recognition and response against vibriosis. These results are similar to the those described in the gills of trout and eels after pathogen infection or salinity changes, respectively, where a high percentage of expressed mRNA transcripts were related to the immune system [46–47]

One of the most differentially regulated transcripts was C-type lectin 1 (*clec1*), which was strongly downregulated by R99 and not by CT285 and therefore may be related to RtxA1_3_ activity. The large family of C-type lectins includes collectins, selectins, endocytic receptors, and proteoglycans, some of them secreted proteins and others transmembrane proteins [48]. In particular, *clec1* has been identified as a receptor expressed by mammal myeloid (dendritic cells [DCs], macrophages and neutrophils) and endothelial (ECs) cells among other immune cells that has a role in T-cell response regulation [49–50]. This result is quite interesting because the few *V*. *vulnificus* cells that can be observed in internal tissues from diseased eels appear closely associated with endothelial cells or with phagocytic cells [22]. Furthermore the toxin has been related to *in vitro* destruction of a wide range of eel and human cells during cell to cell contact occurring in less than 90 min. Observed cell death is produced by apoptosis or pyroptosis depending on the cell type [22, 23, 51–52]. In consequence, the apparent downregulation of *clec1* could be due to death of CDs, macrophages and/or ECs caused by RtxA1_3_. We found evidence of *rtx*-linked cell destruction by necrosis as *casp1*, an important necrosis-related transcript, was differentially upregulated during the first 3 h post infection. At this point *clec1* was clearly downregulated. Caspase-1 is involved in the processing of proinflammatory molecules such as, IL-1β and IL-18 and in programmed cell death mediated by the inflammasome, this process, pyroptosis, being induced by many important pathogens [53]. The hypothesis was partially supported by the rapid increase in *il1r2* transcription observed in the present study that was significantly higher in the gills from R99-infected eels. *il1r2* has been characterized as a decoy receptor responsible for capturing IL-1, produced during inflammation, and reducing IL-1 bioavailability, as a strategy to control inflammation [54].

An alternative or complementary explanation for *clec1* downregulation may be related to antigen presentation by local DCs or macrophages migrating out of the tissue to encounter naïve T cells. Indirect evidence in favour of this can be found in a previous study where we observed *tlr2* and *tlr5*-expressing cells migrating from secondary to primary lamellae during the first hours post-infection with *V*. *vulnificus* [55]. At 12 h post-infection, migration of DCs or macrophages into the tissue contribute to recovering *clec1* and *casp1* basal mRNA levels. However this requires further study to fully understand the dynamics of cellular migration into the gill during bacterial infection.

Our trancriptomic profiling also revealed that another upregulated *rtx*A1_3_-linked mRNA was *cxcr4*. This gene encodes a receptor for stromal derived factor-1, a potent chemoattractant cytokine that modulates stem cell mobilization, inflammatory cell infiltration, and angiogenesis [56]. Interestingly, the Shiga toxin produced by *Shigella* and *Escherichia coli* O157:H7, which cause the hemolytic uremic syndrome in humans, induces a strong over expression of this gene related to endothelial injury [57]. This is a very similar mechanism to that which we suspect is being produced during R99 interaction with eel cells. The authors found that CXCR4 inhibition improves renal function and, ultimately, survival in a murine model [57]. Finally, another upregulated mRNA was *tcr*α, a membrane receptor directly linked to T cells. This finding would corroborate the existence of the recently described lymphoid organ in the gill lamellae of several fish species, site of T-cell aggregation, named inter-branchial lymphoid tissue (ILT) [58–60]. A rapid secretion of specific antibodies in mucus from the gill against *V*. *vulnificus* compared to other mucosal compartments has been previous reported [61], suggesting that the rapid activation of the adaptive immunity molecules may be due to the presence of an ILT in European eel gills.

We also observed evidence of cellular apoptosis and the anti-viral response provoked by *V*. *vulnificus* but in this case independently to the RtxA1_3_ toxin. This was reflected in the upregulation of *caspase-3* and *mx* transcripts, respectively by both strains, although the response was faster in the gills of R99-infected eels. Caspase-3 is a marker for apoptosis or cell suicide and Mx although firstly related to anti-viral response can also represent bacterial-induced cell death [62].

Finally, another putative key molecule involved in response against *V*. *vulnificus* is the myosin regulatory light chain 2 (*mrlc2*) that was upregulated exclusively by CT285. Myosin regulatory light chains regulate contraction in smooth muscle including vascular smooth muscle and have been related with a stimulation of opsonophagocytosis by macrophages [63]. This result may reflect the efficient phagocytosis leading to clearance of the pathogen in the tissue and therefore contributing to host survival.

## Conclusions

We have characterized an immune-enriched reference transcriptome for European eel. 454 sequencing of target libraries constructed from PAMP-activated individual fish was highly successful leading to a high representation of annotated immune-related transcripts. Our results describe a high quality transcriptome that contains significant mRNA diversity and functional relevance for our studies. This resource extends existing transcriptome data and is central to evaluating gene expression associated with immune response in this species. We then designed and validated a custom microarray for studies in the eel that provided a very useful platform to further our knowledge of host-*Vibrio vulnificus* interactions in this endangered species.

From our study we suggest that the processes involved in the early steps of eel vibriosis would be related with cellular destruction of immune and endothelial cells caused by RtxA1_3._ This destruction is not caused by the mutant deficient in the toxin, which in turn would activate phagocytosis by local phagocytic cell populations. To demonstrate the role of the toxin in inducing eel death it will be necessary to analyze the immune response in blood and hematopoietic organs during the first 24 h of infection. Finally, we also found evidence of the presence of an ILT in European eel gills although further experiments will be necessary to identify such tissue.

## Supporting Information

S1 File Characteristics of specific primers used for microarray validation. (XLSX)

S2 File Table A Classification of DEGs by loop analysis after 0, 3 and 12h after R99 or CT285 bath infection. One-way ANOVA followed Tukey’s test with significance value of p<0.05 and no FC cut-off. Table B: Classification of DEGs by relative analysis against handling control group after 0, 3 and 12h after R99 or CT285 bath infection. One-way ANOVA followed Tukey’s test with significance value of p<0.05 and no FC cut-off. Fig A: Venn diagram showing common transcripts differentially expressed along the infection with R99 analyzed by relative analysis approach. Fig B. Venn diagram showing common transcripts differentially expressed along the infection with CT285 analyzed by relative analysis approach.
(XLSX)
